# A novel mutation in the OAR domain of the ARX gene

**DOI:** 10.1002/ccr3.769

**Published:** 2017-01-23

**Authors:** Alejandra Tapie, Natalia Pi‐Denis, Jorge Souto, Alejandra Vomero, Gabriel Peluffo, María Boidi, Martín Ciganda, Nicolás Curbelo, Victor Raggio, Leda Roche, Lucía Pastro

**Affiliations:** ^1^ Facultad de MedicinaDepartamento de GenéticaUniversidad de la República Oriental del UruguayMontevideoUruguay; ^2^ Facultad de MedicinaDepartamento de PediatríaUniversidad de la República Oriental del UruguayMontevideoUruguay; ^3^ Facultad de CienciasLaboratorio de Interacciones MolecularesUniversidad de la República Oriental del UruguayMontevideoUruguay

**Keywords:** ARX, epilepsy, mental retardation, Ohtahara syndrome

## Abstract

Mutations in ARX gene should be considered in patients with mental disability or/and epilepsy. It is an X‐linked gene that has pleiotropic effects. Here, we report the case of a boy diagnosed with Ohtahara syndrome. We performed the molecular analysis of the gene and identified a new missense mutation.

The Aristaless‐related homeobox gene, ARX, is located in the short arm of the X chromosome (Xp21.3). It has been recognized as a cause of mental retardation and epilepsy since its discovery in 2002 1, 2, 3. Several studies have demonstrated high levels of ARX expression in fetal and adult brain, suggesting an important role in neurodevelopment 4, 5, 6.

The ARX gene comprises five exons. The pathogenic sequence variations are frequently located in exon 2, and they involve the expansion of a poly alanine tract 7, 8, 9, 10, 11 and also missense mutations 12. There are few described mutations that involve the OAR domain: a missense mutation in exon 5 of ARX gene (c.1604T>A) resulting in the substitution of leucine to glutamine 13 and a transversion c.1614G>T that produces a lysine to asparagine substitution (p.K538N) 14. Some reported mutations also lead to the complete loss of the domain. For example, a nonsense mutation c.81C>G/p.Y27X 15 leads to domain loss, and an insertion (c.1471_1472insC) results in a frame shift that produce a premature termination at codon 531 within the aristaless domain 16. Two frame shift mutations located in the terminal exon of the ARX gene 17, and some deletions of the c‐terminal region of the protein 18 have been reported. Different mutations in the ARX gene have been widely implicated in several neurologic phenotypes, which underlie its pleiotropic effects in human diseases 11, 18, 19, 20, 21, 22, 23.

The patient we describe (Fig. 1A, III‐3) is the third child of a woman with mild mental disability and the first of a nonconsanguineous couple. The woman had two other children with another partner: a healthy girl and a boy who died at the age of 5 years and who was diagnosed with an epileptic encephalopathy syndrome. The proband was born at term after an uneventful pregnancy and delivery. His birthweight, length, and head circumference were normal. Neonatal seizures were present at 21 days. Brain magnetic resonance imaging (MRI) with spectroscopy was normal. An extensive metabolic investigation was performed, including amino acids and organic acids, and all results were normal. During hospitalization, he presented with several episodes of seizures. Treatment with antiepileptic drugs such as vigabatrin, valproic acid, and phenobarbital was ineffective. The seriate electroencephalograms (EEG) evolved to a burst – suppression pattern compatible with Ohtahara syndrome.

**Figure 1:**
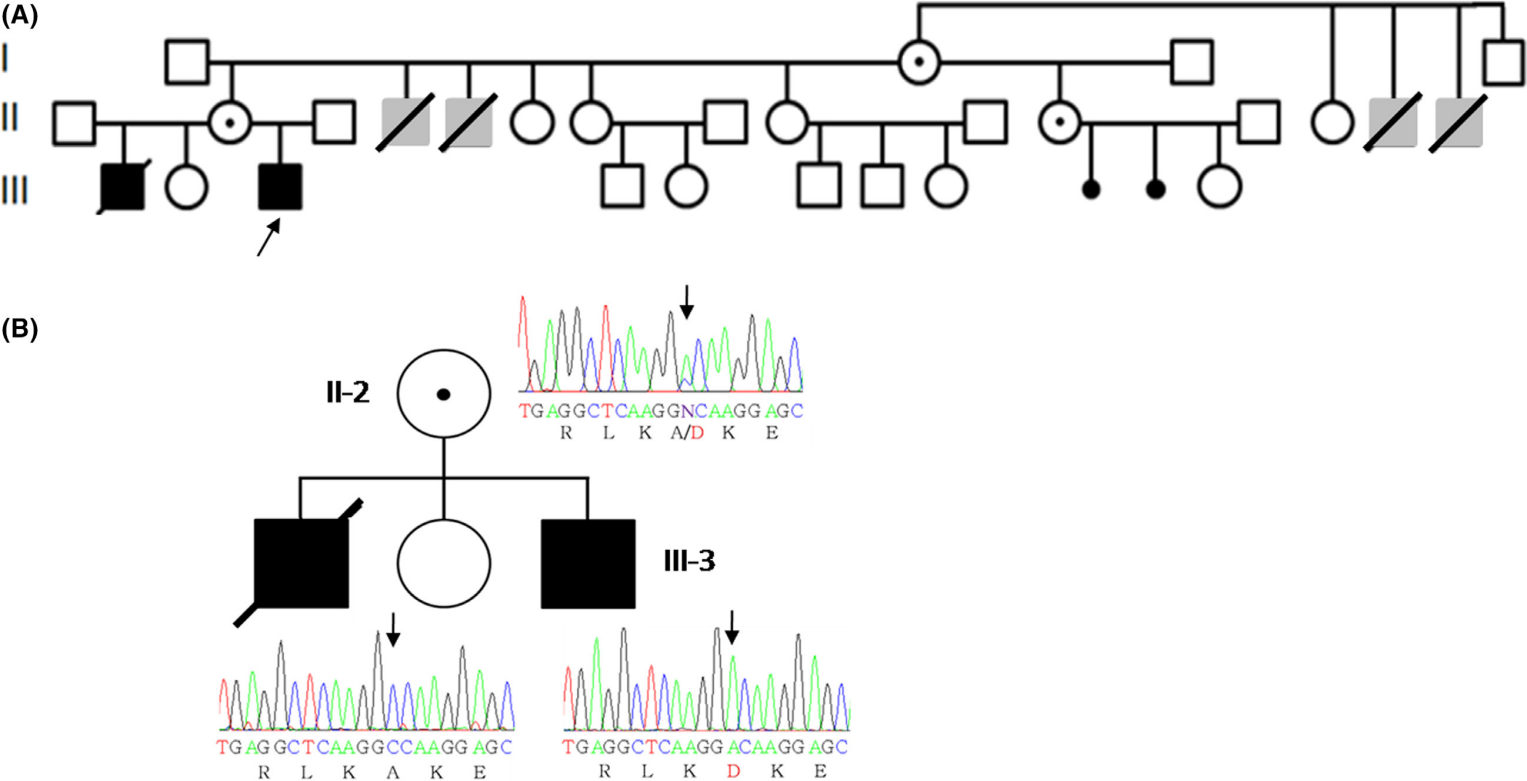
(A) The family pedigree is compatible with an X‐linked inheritance. Proband is designated by an arrow. Symbols in gray represent relatives who are presumed to have been affected. The proband's mother is a carrier, and his sister is a normal homozygote. (B) Sequencing of the proband, his sister, and his mother. Representative sequence traces are shown. Nucleotide change is marked by an arrow. p.A539D amino acid change is shown in red.

His brother (Fig. 1A, III‐1) was brought in for genetic evaluation in 2005 at the age of 5 months. Clinical presentation started at 19 days with seizures and evolved into a severe early infantile epileptic encephalopathy. The EEG displayed hypsarrhythmia, as seen in West syndrome and in the evolution had an EEG with burst – suppression pattern. The patient did not respond well to treatment, remaining with untreatable seizures. He presented with developmental delay and made no progress. A computed tomography scan and metabolic studies were normal. He died at the age of 5 years old, probably as a consequence of the encephalopathy.

After having reviewed the available clinical data and family history, an X‐linked recessive inheritance seemed likely (Fig. 1A) so an ARX‐related disorder was explored.

In the proband, the molecular analysis was performed by sequencing the five exons and exon–intron boundaries of the ARX gene following the Stromme et al. protocol 3.

The mutation analysis was done on extracted DNA from peripheral blood using QIAamp DNA Mini Kit (Qiagen), and the obtained sequences were compared to the ARX reference sequence (NM_139058) by BioEdit software 24.

We found a novel c.1616C>A mutation in exon 5 of the ARX gene (Fig. 1B) in the highly evolutionarily conserved OAR domain. This mutation causes a change of an alanine residue to an aspartic acid residue (p.A539D). This variant is heretofore not described in the Exome Variant Server (NHLBI GO Exome Sequencing Project (ESP), Seattle, WA (URL:http://evs.gs.washington.edu/EVS/) [as of October, 2016]) or in the ExAC database 25, that report mutations in exomes from about sixty thousand persons, this data, strengthen the pathogenic role of the mutation found in our patient.

The patient's mother was found to be a heterozygote carrier. The patient's sister was also studied and turned out to be homozygote for the wild‐type allele (Fig. 1B).

In order to further investigate the role of this change in the patient's phenotype, we performed a bioinformatic analysis. Firstly, we used PolyPhen 26, a tool that predicts the impact of an amino acid substitution on the structure and function of a human protein. The p.A539D mutation described above was predicted to be damaging for protein function with a score of 0.997 (Fig. 2A) suggesting its pathogenicity.

**Figure 2:**
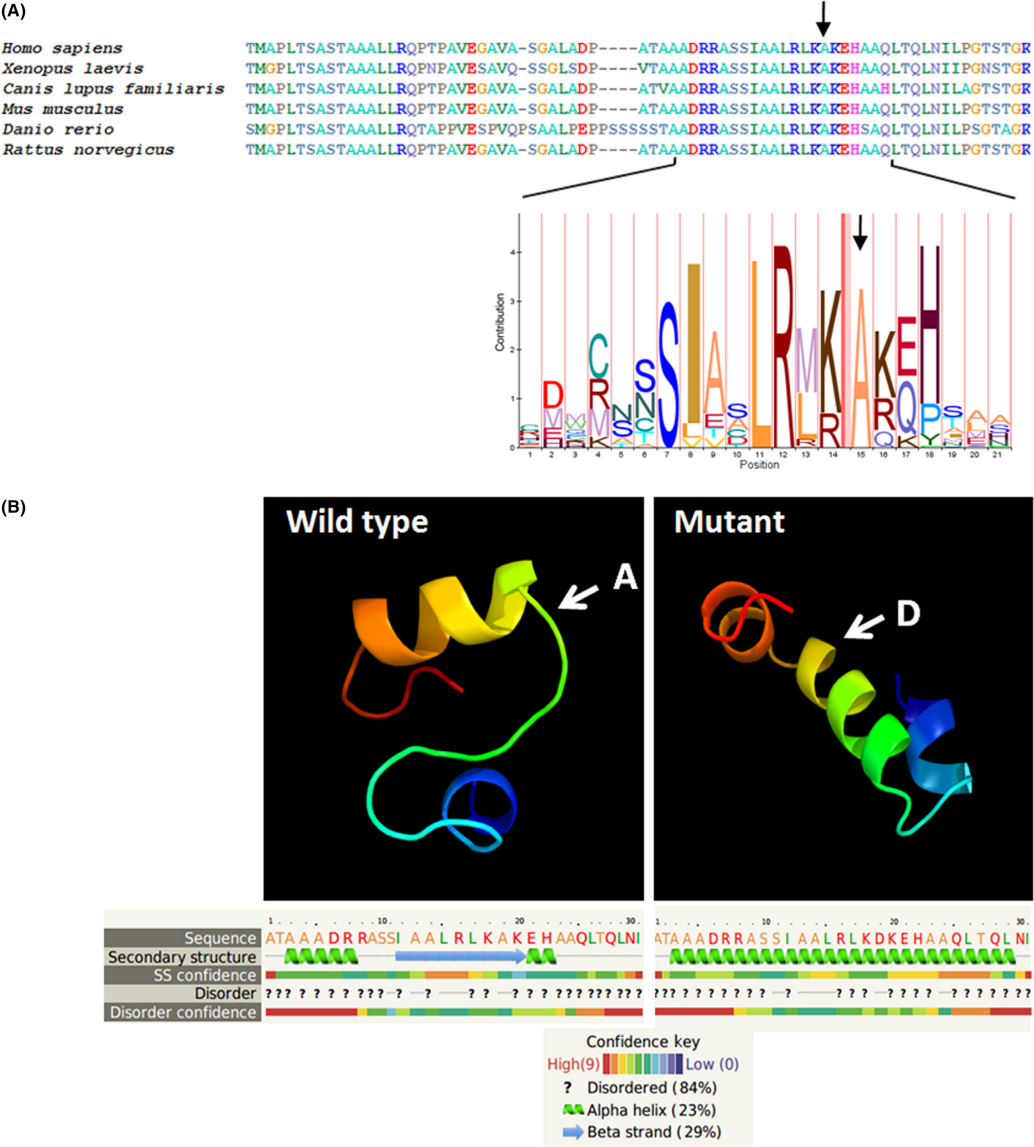
(A) Conservation of OAR domain of ARX. Upper panel. ARX DNA sequences alignment of OAR gene region from vertebrata reported at NCBI database. Bottom panel. OAR HMM model (http://pfam.sanger.ac.uk). (B) Modeling of wild‐type and mutant (p.A539D) OAR domain of ARX protein. The arrow shows the position of alanine (A) and aspartic acid (D) in the wild‐type and mutant protein.

Secondly, sequence alignment of the ARX gene between different species showed that the alanine residue present at position 15 is highly conserved throughout evolution (see Fig. 2A) demonstrating at high degree of conservation for the OAR domain.

Lastly, we modeled the OAR domain using the Phyre 2 software 27. Both the wild‐type and the mutated sequences were used, and we analyzed the amino acid stretch between residues 522 and 552 containing the OAR domain (Fig. 2B). The model predicted that the alanine residue is part of a *β* sheet structure. The amino acid residue substitution produced a significant conformational change affecting the three‐dimensional protein structure. In the mutant model, the aspartate disrupted the *β* sheet structure and produces a new *α* helix structure. This conformational change could induce an important shift in protein function and could be the cause of the disease. It is interesting to note that the transversion c.1614G>T (p.K538N) found by 14 leads to a change in the immediately neighbor amino acid, but the patient's phenotype is remarkable different resulting in ambiguous genitalia and psychomotor delay.

In conclusion, we describe the case of a male child who presented with neonatal seizures and was diagnosed with Ohtahara syndrome. By molecular analysis of the ARX gene, we found a new missense mutation in the OAR domain that could explain the patient's phenotype. The mutation involves an alanine to aspartic acid amino acid residue substitution that is located in an evolutionarily conserved domain and also in a highly conserved position, predicted to be damaging by in silico analysis.

Despite the fact that we do not have a functional analysis of the effect of this mutation, our results strongly suggest this novel mutation to be responsible for the phenotype.

By characterizing a novel variant in a clinical case, this report adds to our knowledge of mutations in the ARX gene that may cause human disease. It also strengthens the case that analysis of ARX should be considered in patients with Ohtahara syndrome and relatives at risk.

## Conflict of Interest

The authors declare no conflict of interest.

## Authorship

AT, GP, AV, NC, MB, and VR: performed the patient diagnostic. LP, NPD, and JS: performed the molecular analysis. LP and AT: analyzed the in silico data. AT, VR, LR, MC, and LP: wrote the paper and discussed the results.
